# Stitchless support-free 3D printing of free-form micromechanical structures with feature size on-demand

**DOI:** 10.1038/s41598-019-54024-1

**Published:** 2019-11-26

**Authors:** Linas Jonušauskas, Tomas Baravykas, Dovilė Andrijec, Tomas Gadišauskas, Vytautas Purlys

**Affiliations:** 1 Femtika Ltd., Saulėtekio Ave. 15, Vilnius, LT-10224 Lithuania; 2 Laser Research Center, Physics Faculty, Vilnius University, Saulėtekio Ave. 10, Vilnius, LT-10223 Lithuania; 3 The General Jonas Žemaitis Miltitary Academy of Lithuania, Šilo Str. 5A, LT-10322 Vilnius, Lithuania

**Keywords:** Applied physics, Techniques and instrumentation

## Abstract

Femtosecond laser based 3D nanolithography is a powerful tool for fabricating various functional micro- and nano-objects. In this work we present several advances needed to push it from the laboratory level use to the industrial production lines. First, linear stage and galvo-scanners synchronization is employed to produce stitch-free mm-sized structures. Furthermore, it is shown that by varying objective numerical apertures (NA) from 1.4 NA to 0.45 NA, voxel size can be tuned in the range from sub *μ*m to tens of mm, resulting in structuring rates between 1809 *μ*m^3^/s and 313312 *μ*m^3^/s at 1 cm/s translation velocity achieved *via* simultaneous movement of linear stages and scanners. Discovered voxel/throughput scaling peculiarities show good agreement to ones acquired with numerical modeling. Furthermore, support-free 3D printing of complex structures is demonstrated. It is achieved by choosing pre-polymer that is in hard gel form during laser writing and acts as a dissolvable support during manufacturing. All of this is combined to fabricate micromechanical structures. First, 1:40 aspect ratio cantilever and 1.5 mm diameter single-helix spring capable of sustaining extreme deformations for prolonged movement times (up to 10000 deformation cycles) are shown. Then, free-movable highly articulated intertwined micromechanical spider and squids (overall size up to 10 mm) are printed and their movement is tested. The presented results are discussed in the broader sense, touching on the stitching/throughput dilemma and comparing it to the standard microstereolithography. It is shown where multiphoton polymerization can outpace standard stereolithography in terms of throughput while still maintaining superior resolution and higher degree of freedom in terms of printable geometries.

## Introduction

With ever growing demand for miniaturized and integrated devices, current fabrication techniques are stretched to their limits. It is mandatory to provide an adequate functionality and a reasonable price of the manufactured structures. This dictates that the fabrication processes have to be extremely precise, flexible and, at the same time, sustain the high throughput. High precision and flexibility are extremely well covered by two photon polymerization (referred to 2PP or TPP) based 3D laser lithography (3DLL)^1^. Over two decades it was applied to a variety of fields: biomedicine^2,3^, microfluidics^4,5^, microoptics^6,7^ and photonics^8,9^. This is due to some inherent strengths of this technique, including possibility to produce nearly unlimited 3D geometry^1,10^, huge array of materials available^11,12^ and capability to produce structures directly on functional substrates^13,14^. Additionally, structures can be made to be in meso-scale. The definition of meso-scale object states that it is a mm to cm sized object with nm-*μ*m features^1^. This allows to achieve nano- and micro-feature enabled functionality in macro structures. Micromechanics are an especially interesting field for 3DLL as it allows assembly-free objects that can achieve various kinds of movement *via* either deformation^15^ or being intertwined^16^. While 3D manufacturing of this kind is becoming increasingly widespread due to complete commercial systems available on the market^17,18^, the adoption of multi-photon polymerization in industry is slow. The reason lies in its point-by-point structuring nature, which can be considered to have relatively low throughput insufficient for the industry^19^. Thus, in order to move it beyond laboratory level use, severe advances in terms of structuring rate need to be made.

This is a well recognized challenge. The variety of ways to increase throughput has been suggested over the years. Parallel manufacturing using several focal points seems as an attractive solution^20,21^. However, in order to implement spatial light modulator (SLM) severe modifications to optical chain have to be performed. Furthermore, all focal points are confined to the working area of an objective, which is rather small for high numerical aperture (NA) objectives (from hundred to several hundreds *μ*m). Thus, only arrays of small and identical objects can be made this way, lessening one of the main selling points of 3DLL - the flexibility of manufacturing. Alternatively, the shape of the voxel can be manipulated. This can be achieved with advanced spatial^22,23^ or spatiotemporal^24^ manipulations of the beam or choosing the objective with appropriate NA. While the first solution is rather complicated, the second one was applied successfully in various works when bigger voxel is advantageous^25,26^. Nevertheless, increasing voxel size is still insufficient in most cases. Fast sample scanning is also required. Due to inherently slow movement or substantial inertia, piezo and linear stages cannot provide it. Scanners, based on freely addressable galvanometrically actuated mirrors, seem as the superb solution due to low nominal inertia, allowing to achieve cm/s translation velocities even when fabricating complex shapes. The problem with scanners lies in limitation induced by the working field of an objective used. A lot of structures with practical potential usage are in meso-scale, making them bigger than the working field of the objective. Therefore, the necessity to print objects segment-by-segment arises, leading to the mechanically^27^ and optically^28,29^ detrimental “stitching”. Hence, scanners are also insufficient to meet all necessities posed by the advances in the field.

In this work on-demand control of feature size and optimization of throughput are presented. It is achieved by employing objectives with different NA in conjunction with linear stage and galvo-scanner synchronization. The presented results give insights into the true effective resolution and throughput tuning range of standard 3DLL setup in terms of the voxel volume and structuring rate. In order to demonstrate the potency of the approach, various micromechanical structures are fabricated. Deformable high aspect ratio cantilevers as well as mm-sized springs are made, their deformation characteristics are tested. Furthermore, intertwined micromechanical objects are made and tested. Finally, an extensive discussion is provided, highlighting the advantages of chosen fabrication strategies and overall 3DLL position in relation to other common optical 3D printing techniques.

## Results

### Resolution on demand with different focusing optics

3DLL is based on non-linear fs pulse absorption in pre-polymer material^30^. Thus, this reaction has a material-specific intensity threshold *I*_*th*_, at which polymerization starts, and upper intensity limit *I*_*d*_, at which material is damaged. The intensity *I* interval between *I*_*th*_ and *I*_*d*_ is called fabrication window for the given set of other exposure parameters (translation velocity (*v*_*t*_), NA, repetition rate (*f*), wavelength (*λ*), pulse duration (*τ*), etc.)^31^. It is important to note that if all other exposure parameters are kept constant and *I* is varied, then fabricated feature size should follow laws of Gaussian laser spot scaling^1^:

1 $$I(r,z)={I}_{0}\frac{{w}_{0}^{2}}{w{(z)}^{2}}\exp (\frac{-2{r}^{2}}{w{(z)}^{2}}),$$

here *r* is the distance from the optical axis and *z* is the distance from the focal plane, *w*_0_ is the spot radius, *I*_0_ - peak intensity at the center of the focus (*r* = 0, *z* = 0). The average laser power (*P*) is usually measured during experiments and can be tied to *I*_0_ using classical formula^1^:

2 $${I}_{0}=\frac{2PT{M}^{2}}{f{w}_{0}^{2}\pi \tau },$$

with *T* denoting objective/system transmission coefficient (for the power measured at the entrance of the focusing optics), *M*^2^ being M-factor or deviation of the real beam from the perfect Gaussian beam and *w*_0_ = 0.61*λ*/*NA* (NA = n *θ* is the numerical aperture of an objective lens defined by the cone angle *θ* of the focusing optics and the refractive index *n* of the material). Gaussian focusing formalism allows to predict voxels to be elongated along longitudinal direction, with line width being *D* and height *L*. Thus, single voxel volume *V* and polymerization rate (i.e. volume structured per time) *R* can be calculated by knowing translation velocity *v* and considering voxel cross section to be near-elliptical^32^:

3 $$V=\frac{1}{6}\pi {D}^{2}L,$$

4 $$R=0.25\pi DLv.$$

**Figure 1:**
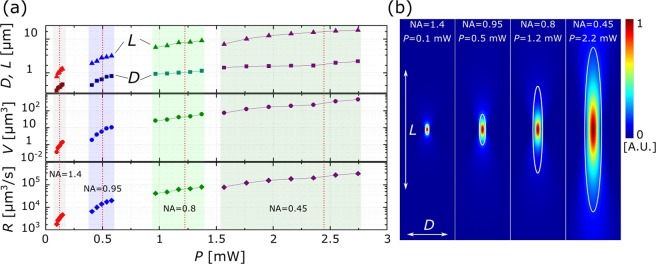
(**a**) Measured transverse (*D*, squares in graph) and longitudinal (*L*, triangles in graph) dimensions of lines fabricated with 1.4, 0.95, 0.8 and 0.45 NA objectives and corresponding voxel volumes (*V*) and structuring rates (*R*). The smooth feature size transition between objectives with different NAs allows to easily choose the fabrication resolution/throughput combination needed for the particular experiment. The colored areas show fabrication windows of each objective. (**b**) Comparison between modeled *I* distributions in focal point with measured line dimensions (white oval). Distributions are normalized to the highest value in each case. Red dashed lines in part (**a**) show what powers were used for the modeling. These are also listed in part (**b**). A good agreement between theory and experiment is evident with *I*_*th*_ = 1.7 ± 1 TW/cm^2^.

Different 3DLL applications might require voxels with either higher resolution or higher volume for faster structuring if feature size is not an issue. Here we apply resolution bridge technique^33^ to measure feature sizes produced with 1.4, 0.95, 0.8 and 0.45 NA objectives [Fig. 1(a)]. *P* (after an objective) is used as the main laser radiation parameter. Other laser parameters: *λ* = 515 nm, *f* = 1 MHz, *τ* = 250 fs. While *P* does not say much about nonlinear process peculiarities it is tied to *I*^30^, it is a lot easier to measure during an experiment and can be used directly in the setup for *P* dependant resolution calibration. Standard hybrid organic-inorganic photopolymer SZ2080 with 1% w.t. photoinitiator Irgacure 369 (IRG) was used for the experiment^31^.

Acquired results allow to estimate that *D* can go from sub-wavelength 0.3 *μ*m with NA = 1.4 while operating near the *I*_*th*_ to 2.18 *μ*m with NA = 0.45 near the *I*_*d*_. Resulting *L* values are respectively 0.77 *μ*m and 18.32 *μ*m highlighting the tendency for *L* to increase a lot more rapidly than *D*. Consequently, *V* varies in the range from 0.036 *μ*m^3^ to 45.51 *μ*m^3^ with *R* being from 1809 *μ*m^3^/s to 313312 *μ*m^3^/s. It means that by virtue of varying NA from 1.4 to 0.45 and *P* in entire fabrication window, it is possible to smoothly choose the *V* and *R* in the range of more than two orders of magnitude. This is due to the maximal feature size with higher NA objective being about the same as the smallest lines with objective with lower NA. However, the care should be taken when fabricating same sized structures with varying objectives as while final feature size might be similar, polymerization degree might differ, influencing mechanical and optical properties of the printed object^15,34,35^.

Finally, the numerical modeling of intensity distributions with each objective was performed using standard Gaussian distribution and M^2^ value of 1.2 (as determined by the producer of the laser). The *P* values were chosen to be above the middle of fabrication window. The values were 0.1, 0.5, 1.2 and 2.2 mW for all the objectives from the highest to the lowest NA. The goal was to determine how accurately one can predict feature sizes before the fabrication using just Gaussian formalism. As shown in [Fig. 1(b)] modeling is relatively close to the measured values, especially at smaller NAs. Also, this modeling showed that it is safe to consider that *I*_*th*_ = 1.7 ± 1 TW/cm^2^ for all the given cases. The ±1 TW/cm^2^ appears due to the fabrication window for each objective being different. Interestingly, in all cases voxel expanded beyond the main focusing volume as the *I* at the border of measured *D* and *L* were in the range of W/cm^2^, i.e. more than ten orders of magnitude smaller. Therefore, it might be considered that when working in the upper part of fabrication window and the polymer is photosensitized, the reaction easily expands beyond high-*I* zone due to radical diffusion^36^ and some defocusing of laser beam by already produced polymeric features. However, with *I* closer to the bottom of the fabrication window, it is offset by the very small volume where radicals are generated and lower defocusing due to smaller difference between refractive indexes of pre-polymer and modified volume^34^. For this reason non-photosensitized^31^ or even photo-inhibited materials^36^ are sometimes used in conjugation to minimal suitable *I* when extra-small features are needed. Finally, voxels expand more in Z direction when NA is reduced. This can be again attributed to some degree of self-focusing, which was shown to sometimes influence laser material processing. Indeed, at special cases, it makes features extremely elongated^37,38^.

### Deformable objects

Polymers used in 3DLL can be considered rigid, unless special elastomer-based materials are applied^39,40^. However, downsizing 3D features and applying special geometries can yield deformable 3D printed structures. This was exploited in micromechanics^16,41^ and metamaterials^15,42^. Yet, objects of this kind demonstrated so far are mostly in the size range of 100 *μ*m, because it fits into the single working field of a standard high NA objective. Furthermore, it means relatively small structure volume and, in turn, potentially fast fabrication.

**Figure 2:**
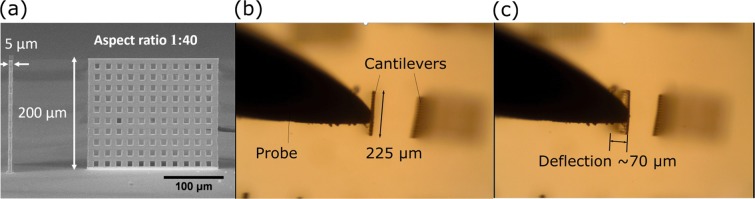
(**a**) A SEM image of cantilever, showing its size and porosity. The aspect ratio of the cantilever is 1:40. (**b**,**c**) show still shots from cantilever bending experiments, demonstrating possibility to bend them up to 70 *μ*m. It can be done extremely violently with metal probe for up to 10 deformation cycles without any adverse effects to the structure.

Here we show the possibility to easily produce high aspect ratio flexible and porous cantilevers. Cantilever in general is elegantly simple, but potent structure for the huge variety of sensors such as AFMs probes^14^ or flowmeters in microfluidics^43^. Indeed, in the later application it could be used to detect flow rates down to nl/min. With such precision it should find applications in high precision drug delivery. 3DLL can be applied for the great effect in fabricating such objects, because they can be printed directly where the measurements have to be performed and their shape can be made to best suite particular application. It is in sharp contrast to currently popular planar lithography techniques that lack this versatility^43^. As an example structure, an upright 200 *μ*m tall, 5 *μ*m wide (aspect ratio 1:40) and 225 *μ*m long cantilevers were 3D printed on glass substrate [Fig. 2(a)]. Additionally, 8.5 *μ*m sized pores were embedded in structures during printing. Pores were needed to achieve higher structure flexibility. It is important to note that with 3DLL pore size and shape can be easily tuned on-demand. Due to these requirements 0.8 NA objective was chosen for the task. In that case voxel was short enough for pores to be manufactured, yet long enough for faster printing in comparison to 1.4 NA objective. While this structure would have fit in the working field of the 20 × 0.8 NA objective (300 *μ*m square) it was still fabricated using stage synchronization as to show that it has not adverse impact on the quality of such a delicate object. Deformation experiments with metal probe demonstrated possibility to bend cantilevers up to 70 *μ*m [Fig. 2(b,c)]. The testing was performed up to 10 deformation cycles as the main goal was to see how polymer cantilever will react to extremely violent bending by metal probe. Even after multiple bending cycles objects retained their original shape, indicating potentially long lifetimes even at extremely adverse experimental conditions. Keeping in mind that 3DLL has a wide array of materials suitable for 3D printing using multiphoton interaction^11,12^, deformable integrated free-form cantilevers seem to be great candidates for the variety of different cantilever-based applications, some of which are already shown to be 3DLL-compatible^14^.

**Figure 3:**
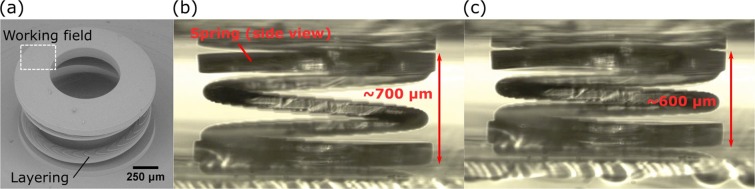
(**a**) A SEM image of 1.5 mm diameter spring with working field of NA = 0.45 objective shown. Layering in the spring is visible. However, despite of that, 100 *μ*m compression (from 700 *μ*m in (**b**) to 600 *μ*m in (**c**), images show side view of the spring) can be carried out 10000 times without any adverse effect to the structure, showing that layering is not a severe problem in such structures.

Next, we moved to the polymeric spring manufacturing. Springs are used in the huge variety of different fields and possibility to print them on demand at any size is an interesting prospect for the technology as it expands possible applications to mm-sized actuators with the necessity for the component to return to its original position and watches. We chose 1.5 mm diameter, 0.7 mm height and single helix two turn design with the helix itself being 0.5 mm tall, 80 *μ*m thick in Z direction and 120 *μ*m in the horizontal plane. With such parameters 20 × 0.45 NA objective was used as the resolution requirements were not high. Continuous writing was also employed to avoid any weak points in the supporting disk or the helix. The print was successful, with spring showing good quality [Fig. 3(a)]. At the same time, the distinction of each printed layer was visible. We will call it “layering”. It appears due to the relatively big slicing step (in this case 9 *μ*m, which is close to 50% of *L*) allowing to maintain maximal possible *R*. The question posed was whether the layering caused mechanical weak points. It was answered by performing deformation experiment with the spring [Fig. 3(b,c)]. Deformation of up to 100 *μ*m (20% of overall height of the helix) was shown to have no negative impact on the mechanical quality of the structure. Furthermore, deformation was repeated for 10000 consecutive times. Even after such huge amount of compressions, spring showed no signs of damage. Therefore, we showed that layering does not have inherently bad effect on the 3DLL made springs and such objects can sustain enough deformation cycles to consider them useful for real world applications.

### Single-step fabrication of intertwined structures

Movement of continuous polymeric structures due to the deformation is widely exploited phenomena^15,16,41,42^. However, next step introduces a special intertwined 3D geometries allowing free and unconstrained movement of different parts of the object after it is printed^44,45^. 3DLL is a superb manufacturing method for such structures because these could be of any required shape, produced at specific sample positions and have precision down to tens of nm^46^. It was used to produce various rotors^44^ or assembly-ready components^45^. Nevertheless, most of these structures are still less than hundred of *μ*m in overall size. Here we present hinge-based micromechanical structures with overall size up to millimeters, while relying on micro-sized features for highly articulated movement.

**Figure 4:**
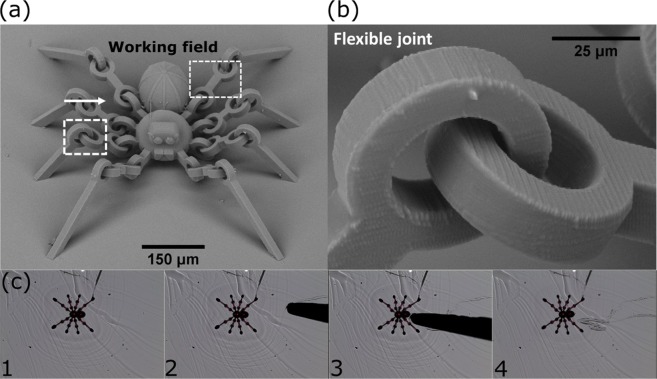
(**a**) An overall view of the movable spider with flexible joint enlarged in (**b**). (**c**) Shows slight movement of spider after it is pushed with metal probe. Movement distance is in tens of *μ*m, best visible at the slight difference at the bend of top left leg. The working field of an NA = 1.4 objective (side length - 125 *μ*m) is also provided, showing that even a single leg fragment would not fit into it if only scanners were applied.

First, a spider structure was printed. It had 8 legs attached to the glass substrate (body was free hanging), each consisting of 3 segments, joined together by micro-hinges. Because SZ2080 is the hard gel, different parts that are not joined together or to the glass substrate can be printed without additional supports. This is somewhat similar to metal 3D printing where unused metal powder acts as the support for features that are being printed. Therefore, the whole manufacturing process is extremely straightforward. No special attachment optimisation or supports are needed creating sharp contrast to cases where liquid resin is used. As structures had to be freely movable after printing, 1.4 NA objective was chosen because it provides the voxel with the lowest and best defined *L*. Nevertheless, *P* of 80% of fabrication window was chosen to exploit highest *R* achievable with 1.4 NA. The distance between separated parts of the hinge were put apart by 5 *μ*m in the model to ensure easy development and to increase the range of movement. The SEM of acquired structure is shown in [Fig. 4(a,b)] showing superb quality of the print. As the working field of the NA = 1.4 objective is a 125 *μ*m square, even a single leg segment would not fit into it [Fig. 4(a)], meaning that scanner-only printing could potentially yield stitching in critical parts of the object. During post-development manipulation, a probe was used to push the spider’s body and proved that it is movable [Fig. 4(c)]. Due to the chosen hinge geometry and angle between the legs, only limited (tens of *μ*m) movement was possible, making it barely visible with optical imaging. Spider broke during more aggressive manipulation.

**Figure 5:**
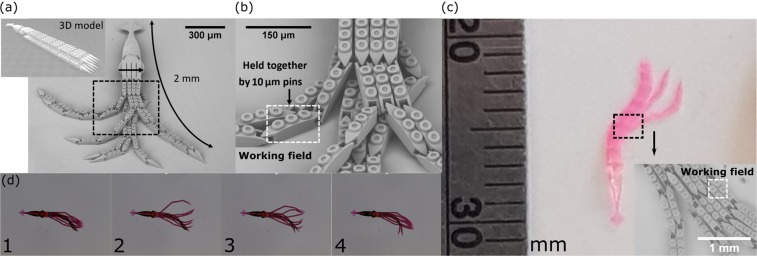
(**a**) An overall view of the movable 2 mm long squid with flexible tentacles with gecko-like suction cups held together by 10 *μ*m pins (**b**). Inset in part (**a**) shows 3D model used for fabrication. (**c**) 10 mm squid printed using the same model, but stretched *via* the software 4.5 times. Inset shows enhanced view of tentacles proving very good manufacturing quality. (**d**) Shows floating of squid tentacles after they are moved by meniscus formed in the thin layer of liquid. Working fields of NA = 1.4 and NA = 0.45 objectives (side length - 125 *μ*m and 350 *μ*m respectively) are also shown in part (**b**,**d**) as white rectangles. It is evident that a single segment of tentacle barely fits in them for both small and big squid.

Next, a structure with more moving parts was modeled - a 2 mm long squid with 8 tentacles, attached to the substrate at the body [Fig. 5(a,b)]. Same 1.4 NA objective was applied with gaps in hinges in Z direction being no smaller than 5 *μ*m. For more convenient printing tentacles were modeled to be straight. The tentacles were held together by 10 *μ*m pins, allowing free and highly articulated movement. Additionally, gecko-inspired suction cup-like structures were modeled on the legs. While they were not tested, it shows that different types of potentially functional geometries at different size scales can be produced on the same object during the same fabrication step. After development the squid was left in the developer and probe was used to create meniscus which in turn moved the structure [Fig. 5(d)]. Tentacles proved to be highly articulated and strong enough to survive the manipulation without breaking. Finally, using 3DPoli software, the same model was stretched by the factor of 4.5 (it is a built-in feature of this software), making the overall printed length 10 mm [Fig. 5(c)]. In order to compensate for the sharp increase in the volume of the structure, objective was changed to 20 × 0.45. Due to the uniform scaling, gap in the model in Z direction grew from 5 *μ*m to 22.5 *μ*m. *L* at the parameters used was 15 *μ*m which was sufficient to avoid different segments of the tentacles being attached to each other during structuring. Printing was successful with single features of tentacles showing superb feature quality [Fig. 5(c)]. It is important to stress that the stretching of the 3D model was done entirely by the software, making it almost hands-free and extremely convenient for the user. Finally, working fields of the NA = 1.4 and NA = 0.45 were plotted on SEM images of both squids [Fig. 5(b,c)]. In both cases even a single segment of tentacle would not fit into it. Thus, we can conclude that continuous writing strategy achieved *via* synchronizing galvo-scanners and linear stages is imperative for defect-free 3D printing of meso-scale mechanical structures.

## Discussion

Changing NA to achieve application-required voxel size is a popular technique applied in 3DLL for years by various groups^2,7,9,26^. However, in most cases the application-specific voxel size is found and then used with minimal explanation on what feature sizes could be expected while changing NA in a relatively wide range with the same material and the same setup. The information on full voxel size calibration for several objectives at once is severely lacking in the literature. Also, while there are some works trying to tie voxel size dynamics with some theoretical models^30,47^, in most cases the theory is rather complicated and requires some very specific knowledge about the material (for instance, second order absorption cross-section *σ*_2_) and the process (for example, order of nonlinearity), rendering it too complicated to be used on daily basis. In this article we present full and continuous voxel size calibration and tie it with very simple Gaussian focusing modeling. While more complex electrical field distributions are present at focal point when focusing with objectives having NA > 0.2^48^, Gaussian-based calculations yield the result which differs from the real voxel sizes less than 10%, which is completely acceptable for approximate calculations. Furthermore, we show that finding the fabrication window with one objective allows calculating the structuring-suitable *I*, which can be reverse calculated to *P* to find structuring parameters with other NAs. Only basic laser system parameters (*P*, *T*, *λ*, *f*, *τ*, NA) and very simple arithmetic operations are needed for it, making it extremely simple to use on daily basis. What is more, the feature sizes presented here mimic very well what was found in other works. For instance, when high NA immersion objectives were used with materials like acrylate-based SR500^49,50^, hybrid organic-inorganic ORMOCER^51,52^, lithography-oriented SU8^52^ or 3DLL specific IP-Dip^47^, *D* was well into hundreds of nm with aspect ratio 3.5 ±1. This is very close to values measured here with NA = 1.4 objective and photosensitized SZ2080 material: *D* = 0.3–0.45 *μ*m with aspect ratio at around 2.5–3. The only exceptions can be expected with materials having heavily modified photosensitivity^35,36^. The agreement continues with lower NAs as well. For instance, when 20 × 0.8 NA objective was used to structure bio-derived polymer *D* = 1–1.4 *μ*m^53^, which is relatively close to the results presented here (*D* = 0.9–1.2 *μ*m). At NA = 0.55, *D* varied in the range of 0.8–3.4 *μ*m using ORMOCER material^54^. The closest objective employed in this study was NA = 0.45, where *D* = 1.4–2.18 *μ*m, which coincides well to interval acquired with ORMOCER. The overall bigger *D* interval might be tied to potentially wider fabrication window of ORMOCER. However, aspect ratio is very close in both cases (around 10 for ORMOCER and around 9 for SZ2080 with 1% w.t. IRG). With NA = 0.3 and SU8, *D* goes from 1 *μ*m to even 6 *μ*m^25,55^ - a result that can be expected by considering *D* increase dynamics with decreased NA visible in Fig. 1(a). The main difference in all of these studies lies in *P* used to achieve the same feature sizes. It varies from tenths of mW to more than 10 mW (especially at lower NAs). Such a huge discrepancy is a result of differences in laser system parameters, namely *τ* (from tens to hundreds of fs), *λ* (most popular ones: 780/800 nm and 1030/1045 nm with appropriate harmonics) and *f* (from hundreds of kHz to tens of MHz). However, when recalculated to *I*, the values mostly fall in TW/cm^2^ range. Thus, the results provided here can be considered as a very generalized guideline for approximately evaluating what feature sizes can be achieved with various objectives. What is more, it is not limited to just tested laser system and material.

There were numerous attempts to apply 3DLL for microrobotics^56,57^. Straightforward manufacturing of intertwined structures puts an interesting prospect in the field, as these hard objects can act as a “bone” structure for a complex microrobot that could then be propelled by some other means. Therefore, due to 4D structuring capability of 3DLL^3,41^, elastic materials could be printed on such hard parts, creating bio-mimicking system. Having in mind fast developments in the field of optostructurable artificial muscles^58,59^, it points to a very powerful future application of the technology. It is especially true keeping in mind that, as shown in this work, sub-cm stitchless 3D meso-printing with on demand resolution is easy to realize in current state-of-the-art 3DLL systems.

Stitching is quite controversial topic in the field right now. The problem lies in somewhat different view by various groups to what exactly should and should not be considered a stitch. While it mostly refers to seems between segments fabricated using scanners, it is important to realize that on the technical level it can be considered to go beyond it. Technically, all serial production, to some extent, is based on it. In fact, pulsed laser-based point-by-point manufacturing has multiple levels of stitching. First, single pulses of laser join together to form a line. Then, lines are joined together. Finally, layers are formed one by one. However, while all of these joining points can be considered “stitches”, practically it has minimal impact. When fs laser is used for 3DLL, distance traveled between pulses rarely exceeds 10% of a laser spot, forming continuous line^32^. Line and layer overlap depends on the application and has to be in accordance with the functionality of an object^60^. Spring demonstrated in Fig. 3 is a good example, because it has clear layering (i.e. “stitching” in Z direction) but it has no negative impact on the mechanical functionality of an object. This would not be acceptable in microoptics^7,32^.

**Figure 6:**
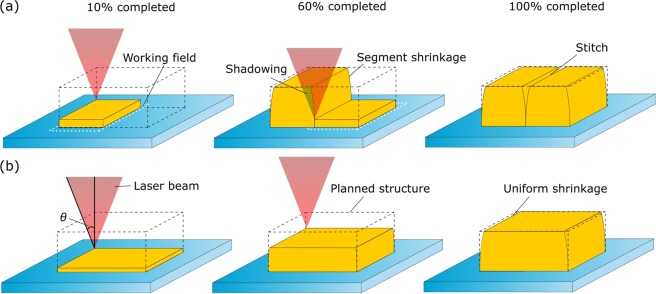
Schematics explaining how shadowing and shrinkage influence the severity of stitching defects. Simple bulk structure will be shown at 10%, 60% and 100% completion with the pre-fabrication model denoted in black dashed lines. Using only scanners two segments have to be produced due to the limited working field (shown as white doted lines). (**a**) After the first segment is produced, it is blocking some of the laser beam needed to properly structure the bottom of the next segment. This effect is called “shadowing” and is marked as green dashes on the laser beam. The severity of shadowing depends on *n* mismatch between polymerized and unpolymerized resin, height of the first segment and particular objective (as NA = *n*sin *θ*). Furthermore, each segment shrinks independently from the previous one making their connection harder. In the continuous writing case (**b**) layers produced after each other do not obstruct the light and the shrinkage happens uniformly. Thus, while sides of the structure might be somewhat distorted depending on how much the material shrinks, overall structure has a lot better quality. Also, if all other parameters are chosen appropriately, structure’s quality is not inherently influenced by such parameters as *n* mismatch or the NA of the objective.

Thus, what is the difference between all of the discussed cases and stitching between segments made by single working field? The answer lies in the material shrinkage and shadowing of laser beam by already produced structure [Fig. 6]. While there are materials with minimal shrinkage, it is rarely smaller than 1–2%^61^. Therefore, when a segment is produced, it shrinks in the volume of pre-polymer deviating from the desired shape. This happens even if the formed segment is attached to the glass substrate. Such deviations in segments start to accumulate and become visible in the form of stitches. If the continuous scanning is applied, the whole layer shrinks together. It still results in some sort of deformation, but in that case it is distributed more equally in the structure and is not concentrated at the edges of segments. Furthermore, when high enough segment is produced, it has the potential to block part of focused laser light when other segment is made close by. This effect is called “shadowing” and was observed when using 3DLL to integrate structures to channels^62^ or producing different parts of an object in consecutive manner^63^. Additionally, *n* mismatch between polymerized and unpolymerized resin starts to play a role in the severity of the effect. It is in sharp contrast to continuous writing where *n* of material before and after structuring does not influence the quality. The process is also more pronounced in objectives with higher NAs, because then *θ* is increased, resulting in more of a focusing cone being blocked by the surrounding segments while scanning. Overall, it means that additional steps have to be taken either designing the structure or optimizing the manufacturing algorithm in order to avoid these effects^64^. While it can sometimes be done in relatively simple and generic way, it is still an additional operation that has to be performed in otherwise relatively straightforward fabrication process. Finally, stitching can be realized by making all the segments of a single layer, i.e. avoid block stitching and shadowing. However, then, the inertia of mechanical axes become a limiting factor and the whole process starts to resemble continuous writing. Indeed, during synchronized manufacturing the whole layer is produced in one go with minimal segmenting and with maximal possible speed. Thus, shadowing is completely averted and shrinkage is distributed through the whole structure.

**Figure 7:**
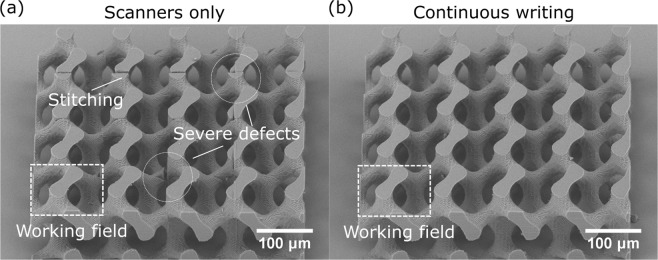
A 3D gyroid structure printed with scanners (**a**) and using continuous writing *via* synchronized linear stages and galvo-scanners (**b**) at *v* = 1 cm/s. Objective - 63 × 1.4. NA, working field - 125 *μ*m square is marked as white dashed rectangle. Clear stitching is visible in part (**a**) with some defects resulting in near breaking of the objects. These appear due to the segment shrinkage and shadowing of already made parts of the structure. It completely compromises 3D gyroid structure because it should be extremely mechanically strong. In contrast, using continuous writing, there are no such defects and the structure is of superb quality.

But are there general guidelines when inter-segment stitching becomes unacceptable? The answer depends whether the stitching compromises the functionality of an object. Indeed, scanners still offer an advantage of faster and simpler fabrication if mass production of relatively small objects is required or stitching-associated defects are not an issue. However, in some cases stitching can completely compromise the functionality of an object. This is extremely relevant for optics^28,29^ and for micromechanics^27^. In the latter case stitching would create mechanical weaknesses in the structure. In order to demonstrate that an example structure of 3D gyroid was made. In the stitched case clear cracks are visible [Fig. 7(a)], undermining the main idea behind 3D printed gyroid structure - extreme mechanical resilience with minimal weight^65^. This would also apply to all other objects tested in this work. Therefore, stitching has the tendency to severely undermine meso-scale structures. Stitches are nm-*μ*m level defects that potentially prevent structures from having some highly desirable functionality. On the other hand, continuous writing provides good quality structure that does not have any inherent manufacturing-induced defects [Fig. 7(b)]. Thus, usage of synchronized positioning for continuous writing is a powerful way to supplement scanners. This is especially true, because each system equipped with both scanners and linear stages can always be used in unsynchronized manner, meaning that the user can freely choose between using just scanners for higher speed or scanners synchronized with linear stages for higher quality of meso structures.

Presented feature size and polymerization rate *R* results allow to compare 3DLL to stereolithography (SLA). Thinking in a conventional way, it should be obvious that 3DLL is superior in terms of resolution and SLA can make bigger things faster. However, as this work showed with relatively small NA, 3DLL *R*_3*DLL*_ can be relatively high (up to 313312 *μ*m^3^/s at *v* = 1 cm/s). In comparison, micro-SLA can yield feature size of *D*_*SLA*_ = 7.5 *μ*m and *L*_*SLA*_ = 20 *μ*m at *v* = 500 mm/s^66^. If we apply the same *R* formula, we get that *R*_*SLA*_ = 58875000 *μ*m^3^/s. So far nothing unexpected - due to bigger voxel and higher *v*, *R*_*SLA*_ exceeds *R*_3*DLL*_ by two orders of magnitude. However, when dealing with SLA one must not forget the time needed to recast polymer after each structure layer is made^1^. This means that after each layer is made there are approximately 2–10 seconds down time^67,68^. Indeed, this is one of the main bottlenecks in SLA, sometimes taking up to 90% of overall printing time^68^. Exact value depends on the viscosity of the material and required layer thickness^68,69^ and can be expected to differ in some special cases. In contrast, the down time between layers is not present in 3DLL where all material drop is present from the beginning of the fabrication. In order to demonstrate the implications of this, let’s consider fabrication of 100 × 100 × 100 *μ*m cube. For the simplicity we will not consider voxel overlap for this example, although it is very important parameter during 3DLL manufacturing. If we only consider the volume fabrication time, 3DLL should fill such volume in 3.19 s, SLA in 0.02 s. However, keeping in mind that *L*_*SLA*_ = 20 *μ*m, 5 layer recasts will be needed which will last 25 s total if 5 s down time is considered. In such case, 3DLL will outpace SLA as it never stops. Interestingly, if the same volume was fabricated with just one layer recast, the overall printing time would be almost the same. Thus, it means that SLA is benefiting from wide and low structures with minimal layer number, while in 3DLL case there is no difference. However, if the structure size is increased to 1 × 1 × 1 mm, even considering 50 layer recasts SLA will be faster than 3DLL 267 s *vs* 3192 s). Therefore, currently, 3DLL can have a throughput edge only in sub-mm printing. The situation is not much different with dynamic mirror device (DMD) based SLA printers. On one hand, these 3D printers manufacture the whole layer in one exposure, eliminating scanning^1^. On the other hand, the exposure time of one layer can go from several (for optimized materials) to tens (for unoptimized polymers, for instance bio-derived) of seconds^70^, adding to layer recast times. In contrast, with 3DLL, due to very sharp and relatively aggressive energy introduction mechanism, even unoptimized materials can be structured relatively fast^53^. Finally, all polymers designed to be used in SLA and DMD have to be liquid for already mentioned polymer layer recast. 3DLL can employ both liquid and hard materials. In the latter case material can act as a support (somewhat similar to unstructured powder in selective laser melting^1^) eliminating the need of supports. This is another area where 3DLL is superior to SLA/DMD. Therefore, it can be considered that currently 3DLL with low NA objectives can actually outperform the throughput of SLA and DMD 3D printing at sub-mm structure manufacturing while still maintaining the edge in terms of resolution and applicable geometries.

Finally, one might expect 3DLL throughput to grow in the future. Here, making voxels bigger is not an option due to the loss of one of the key advantages of 3DLL – very well-defined 3D structures. An option would be to increase translation velocity up to m/s. Indeed, with translation velocity reaching 1 m/s and considering the same voxel dimensions *R* = 31331200. Then, 1 mm^3^ cube can be fabricated in around 32 s, outpacing previously considered SLA where total fabrication time was 267 s. Even if layer recast down time is not considered, the result is close as pure printing time with SLA in that case is around 17 s. Such *v* can be considered completely realistic for specialized 3DLL setups even now. Indeed, it was already shown that modern positioning can support such translation velocities if relatively low quality structures are acceptable (for instance, scaffolds for cell growth^71^). However, relatively slower cm/s level *v* is still required for highly complex high-quality structures. Further advances in hardware and software are needed to achieve better printing quality at *v* > 1 m/s. New ways to project laser beam might be applied in the future for 3DLL, like polygon scanners^72^ or acousto-optical deflectors^73^. They might pose some limitations like limited flexibility for polygon scanners or temporal ultrashort pulse distortion in the case of acousto-optical deflectors. However, the possibility to achieve translation velocities well into tens of m/s is extremely attractive, potentially transforming 3DLL into technology which is both: more precise/flexible and faster than the standard single photon absorption-based SLA/DLP.

## Methods

SZ2080 photopolymer was chosen for this work as it exhibits low shrinkage^61^, good mechanical stability^31^ and low optical absorption for the whole visible part of the spectrum^74^. It was photosensitized with 1% w.t. photoinitiator Irgacure 369. To allow easier observation under optical microscope it was also mixed with rhodamine^32^ for some of the micromechanical structure fabrication. Mixing rhodamine and SZ2080 had no significant impact to voxel size or mechanical properties of the material making its usage simple and straightforward. Samples were prepared by drop casting material on the glass slide and then pre-baking them on 50 °C for 1 hour. Development was performed in isobutyl methyl ketone for up to 45 minutes. Then, they were dried in ambient conditions. Characterization of the samples was carried out using SEM TM-1000 (Hitachi), various optical microscopes and mobile phone cameras. Keep note that they appear as rectangles in SEM images captured at 45° angle. More information about it can be found in previous article dealing with feature sizes at SEM images at various angles^33^.

**Figure 8:**
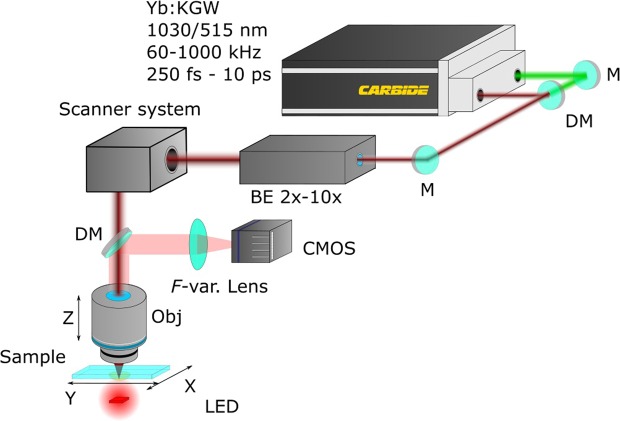
Schematics of “Laser Nanofactory” used in this work. Markings: M - mirror, DM - dichroic mirror, BE - automated beam expander, *F*-var lens - variable focal distance lens, Obj. - objective.

Sample fabrication was performed using “Laser Nanofactory” (Femtika) setup [Fig. 8]. The main light source in this setup is femtosecond laser “Carbide” (Light Conversion), outputting either fundamental (1030 nm) or second harmonic (515 nm) radiation at repetition rates in the range of 60–1000 kHz and pulse duration between 250 fs and 10 ps. Average power is controlled with acousto-optical element integrated in the laser as well. Laser light is then guided to automatic beam expander with magnification range from 2x to 10x. This tunability is needed to precisely match laser beam diameter to entrance aperture of arbitrary objective. Finally, it is directed to the scanner system (AGV-10HPO (Aerotech Inc.)) and then to a focusing objective. Scanners work in tandem with linear stages (ANT130XY-160 (Aerotech Inc.) for XY and ANT130LZS-060 (Aerotech Inc.) for Z axis) allowing synchronized movement and, in turn, stitch-free positioning. User in such system only inputs the general 3D model and/or manually programmed trajectory of writing. It is done in proprietary software 3DPoli. The distribution of movements between linear stages and scanners is done automatically by the controlling software reducing the workload of a user and assuring superb optimization. The idea behind it is taking the whole movement and, depending on what acceleration rates are possible with linear stages and scanners, dividing it between them. First ones get long “slow” movements, while galvo-scanners perform short “fast” movements^75^. For this reason linear stages and galvo-scanners are moving simultaneously all the time, hence the term “continuous writing”. While 5 axis synchronization and simultaneous movement is possible (3 linear stages and 2 scanners) with such system^32^ allowing true 3D writing trajectories, in this work all structures were fabricated in layer-by-layer fashion using STL models. It was done for simplicity reasons. Whole process is imaged through built-in visualization system, employing CMOS camera, variable focal length lens (needed for imaging with different objectives) and a red LED. Objectives tested in this work: 63 × 1.4 NA (Zeiss), 40 × 0.95 NA (Zeiss), 20 × 0.8 NA (Zeiss) and 20 × 0.45 NA (Nikon). All the components in the system are controlled with 3DPoli software (Femtika). Working fields for these objectives were squares with side lengths of 125 *μ*m, 200 *μ*m, 300 *μ*m and 350 *μ*m respectively.
